# Analysis of Ki67, HMGA1, MDM2, and RB expression in nonfunctioning pituitary adenomas

**DOI:** 10.1007/s11060-016-2365-9

**Published:** 2017-03-02

**Authors:** Xiaohui Yao, Hua Gao, Chuzhong Li, Lijuan Wu, Jiwei Bai, Jichao Wang, Yangfang Li, Yazhuo Zhang

**Affiliations:** 1 Key Laboratory of Central Nervous System Injury Research, Beijing Neurosurgical Institute, Capital Medical University, Tiantanxili 6#, Beijing, 100050 China; 2 Shanxi Provincial People’s Hospital, Taiyuan, Shanxi China; 3 Neurosurgical Department, Beijing Tiantan Hospital, Beijing, China; 4 Department of Neurosurgery, Xinjiang Uygur Autonomous Region People’s Hospital, Xinjiang, China

**Keywords:** Nonfunctioning pituitary adenoma, Tissue microarray, Recurrence, Regrowth, HMGA1, MDM2

## Abstract

Nonfunctioning pituitary adenomas (NFPAs) are the most prevalent type of pituitary macro-adenoma. Clarifying the relationship between NFPA markers and disease progression or recurrence could provide a basis for administration of adjuvant treatments. The present study examined the expression levels of high-mobility group (HMG)A1, Ki-67, mouse double minute 2 homolog (MDM2), and retinoblastoma (RB)with respect to NFPA recurrence. Immunohistochemistry was carried out using antibodies to Ki-67, MDM2, HMGA-1, and RB on tissue microarray slides of a cohort of 35 paired NFPA samples of primary and recurrence/regrowth tumors. Based on postoperative magnetic resonance imaging data, tumors were classified as recurrence (n = 20) included primary and recurrent tumors or regrowth (n = 15) included primary and regrowth tumors, which are paired. Protein expression was classified as negative or positive according to the H-score method and was analyzed with respect to clinical and pathological findings. MDM2-positive cases accounted for11/20 primary and 19/20 s recurrent tumors (χ^2^ = 8.533, P = 0.003), and 9/15 primary tumors and 15/15 s regrowth tumors (χ^2^ = 7.5, P = 0.006). MGA1-positive cases represented 9/20 primary tumors and 16/20 s recurrent tumors (χ^2^ = 5.227, P = 0.022), and 4/15 primary tumors and 12/15 s regrowth tumors (χ^2^ = 8.571, P = 0.003). There was no statistically significant difference in Ki-67 expression between primary and second recurrent/regrowth tumors although theKi67 labeling index was higher in the latter groups. RB was highly expressed in all groups with no significant difference between them. HMGA1 and MDM2 were more highly expressed in recurrence/regrowth cases of NFPA than in primary NFPA. HMGA1 and MDM2 are biomarkers and potential drug targets for NFPA treatment.

## Background

Nonfunctioning pituitary adenomas (NFPAs) are the most prevalent type of pituitary macroadenoma, accounting for 25–35% of all cases [1]. Although they are generally benign, many invade the sphenoid, cavernous sinus, or dura mater and may be incompletely removed by surgical resection. The remnant tumor can potentially regrow since residual cells retain their ability to proliferate, necessitating a second therapeutic intervention [2]. Most cases of recurrence occur within 5 years after surgery [3]. Owing to the high rate of long-term recurrence, patient prognosis is not always favorable [4]. The main therapeutic option for NFPA is surgical and there are currently no pharmacologic treatments available. A variety of histological biomarkers for NFPA have been investigated for their relationship to invasiveness and tumor recurrence, including the proliferation marker Ki-67, cellcycle-related factors such as p27 and galectin-3, and other molecules such as p53,O-6-methylguanine-DNA methyltransferase, and matrix metalloproteinase 9 [5–7]. However, given the absence of reliable serum markers for detecting residual tumor cells, the decision on whether to recommend additional intervention is typically made based on postoperative imaging.

The high-mobility group (HMG)A family of proteins has four members; one of these, HMGA1, plays a critical role in cancer progression, development, and metabolism, among others [8]. HMGA proteins are expressed at low levels in normal adult tissues and cells, but is upregulated in many tumors, neoplastically transformed cells, and embryonic stem cells [9]. HMGA overexpression is associated with poor prognosis due to metastasis [10]. HMGA1 has been linked to pituitary tumor progression, consistent with its critical role in cell cycle regulation [11]. HMGA2 is associated with the retinoblastoma (RB)-E2F pathway in pituitary cell proliferation [12]; RB protein is also involved in cell cycle control. Unlike HMGA2, the role of HMGA1 in pituitary tumorigenesis remains poorly understood. The expression of HMGA1 suggests a link to a secondary event in pituitary gland tumorigenesis [11].

Mouse double minute 2 homolog (MDM2) plays a critical role in the regulation of the tumor suppressor protein p53 [13]; MDM2 overexpression inhibits p53 activation, leading to evasion of the cell cycle checkpoint and carcinogenesis [14, 15]; it is also overexpressed in many human malignancies [16]. Ki-67 is a reliable cell proliferation marker in immunohistochemistry (IHC) used to assess tumor progression in routine histological analyses [17]. Although it is routinely applied to pituitary adenomas, its prognostic significance remains controversial [18, 19].

Tissue microarrays (TMAs) are assembled from many tissue samples in a single paraffin block to increase the throughput and performance of molecular profiling studies in tumors, and can reduce experimental variables and conserve tissue samples. In this study we used TMAs to evaluate the expression of HMGA1, Ki-67, MDM2, and RB in 35 paired NFPA cases of primary and recurrent/regrowth tumors to identify a suitable marker for NFPA progression.

## Materials and methods

### Patients

This retrospective study included 35 patients at the Beijing Tiantan Hospital, Captital Medical University, who underwent transphenoidal or transcranial operation from January 2008 to December 2013.Tumors were all NFPAs according to the 2007 WHO histologic classification. According to Knosp classification and records of operation, NFPAs included non-invasive and invasive tumors (Table1). There were classified into recurrence group included primary and recurrenttumors which are paired and regrowth group included primary and regrowth tumors which are paired. Recurrence was diagnosed in 20 patients when a new tumor was histologically confirmed, Regrowth in 15 patients was diagnosed by the growth of a residual tumor, which was evaluated by a neurosurgeon and two neuroradiologists who were blinded to the patient’s characteristics (see in Sheme 1). The study was carried out according to an institutional review board-approved protocol, and written; informed consent was obtained from all patients prior to surgery.

**Scheme 1:**
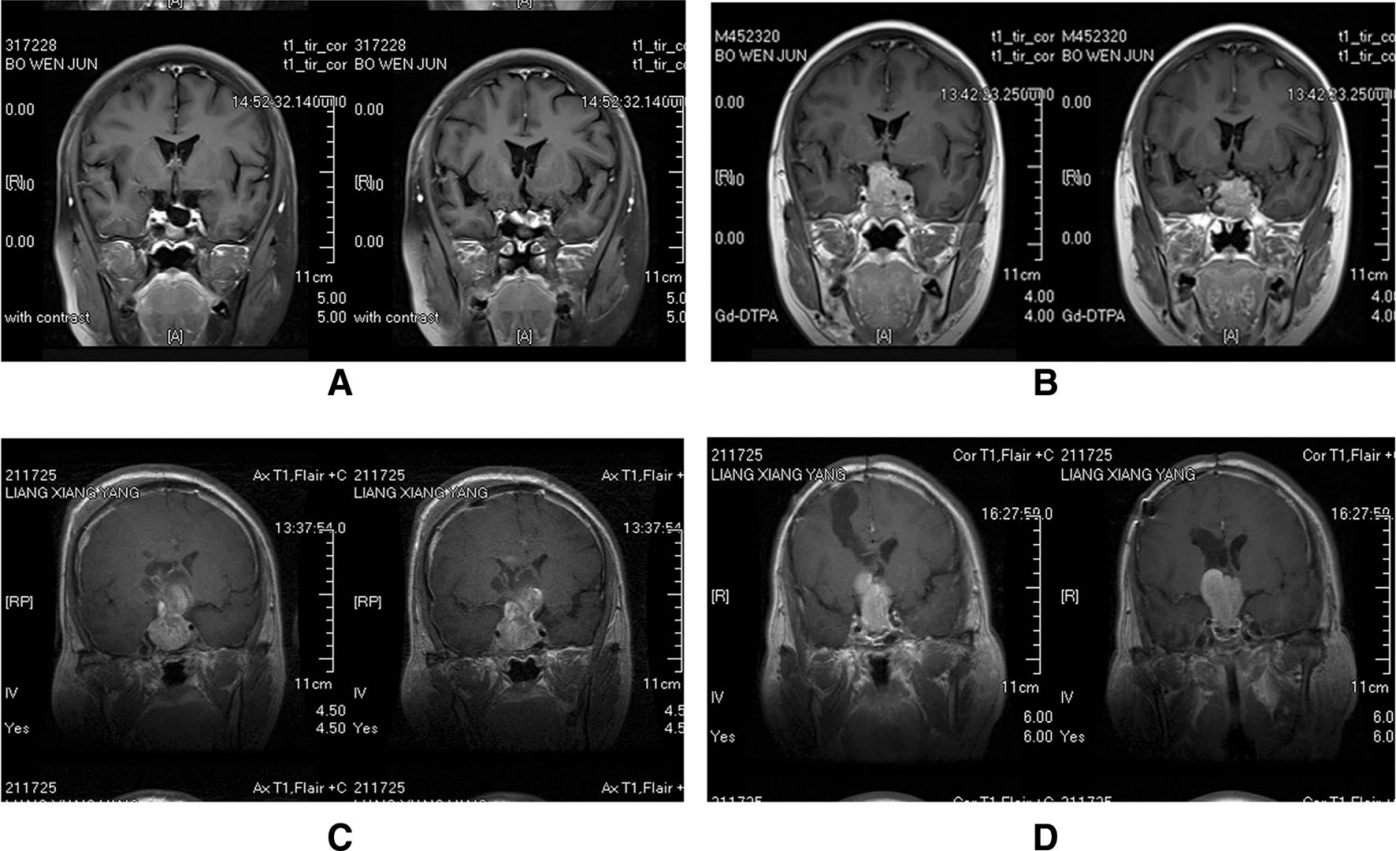
**a** First postoperative MR image demonstrating complete tumor removal in a 30-year-old women. **b** MRI scan obtained 24 months after surgery shows a distinct tumor recurrence in the sellar. **c** First postoperative MR image demonstrating incomplete tumor removal in a 41-year-old men. **d** MRI scan obtained 58 months after surgery shows a distinct tumor regrowth in the sella

### TMA construction

Formalin-fixed, paraffin-embedded tissue blocks were stained with hematoxylin and eosin (H&E). Three core biopsies with a diameter of 2.0 mm were transferred to TMAs using the Leica BOND-III fully automated arrayer (Leica Biosystems, Wetzlar, Germany). The core samples were randomly ordered and the pathologist was blinded with respect to their location on the TMA slides. TMAs were cut into 4-µm sections using a serial microtome and placed in a water bath at 50 °C; the sections were transferred to positively-charged glass slides, deparaffinized, and rehydrated through a graded series of alcohol with water as the final solution. Slides were dried at room temperature for 24–48 h and stored in a freezer at −80 °C until use. To minimize loss of antigenicity, sections were processed within 1 week of cutting [20].

### IHC

Tumor content and quality were evaluated in TMA slides by H&E staining. Antibodies against the following proteins were used under the indicated conditions: Ki67 (Abcam, Cambridge, UK), protocol F, with 20 min of epitope retrieval (ER) and 15 min of heat-induced epitope retrieval (HIRE); MDM2 (Abcam), with 3 min of ER and 30 min of HIRE; HMGA1 (1:3000; Abcam),with 20 min of ER and 15 min of HIRE; and RB (1:500; Abcam) with 30 min of ER and 30 min of HIRE. Bond Polymer Refine Detection (DS9800; Leica Biosystems) was used to detect the primary antibodies. The slides were scanned as digital images using Aperio AT2 (Leica Biosystems). The intensity of staining was calculated by two neuropathologists who were blinded to the patient’s clinical and radiologic information.

### TMA scanning and image analysis

Antigen labeling index (LI) was determined by counting the number of positive cells in a total of 1000 tumor cells in the maximally stained region at high magnification (400×). The staining intensity was stratified on a scale of 0–3 (0 = no staining, 1 = weak, 2 = moderate, and 3 = strong). An H-score was obtained by multiplying the staining intensity with a constant to adjust the mean to the strongest staining [score = 1.0(%weak) + 2.0(%moderate) + 3.0(%strong)].

### Statistical analysis

The χ^2^ test was used to assess the significance of associations among HMGA1, Ki67, MDM2, and RB expression and clinical parameters. Differences were considered statistically significant at P < 0.05. Analyses were carried out using SPSS v.19.0 software (IBM, Armonk, NY, USA).

## Results

### Characteristics of the cohort

We selected 35 consecutive patients diagnosed with NFPA for whom primary and recurrent; primary and regrowth tumor specimens were available, which are paired. Visual disturbance (23/35, 65.7%) was the most common clinic symptom, followed by headache (22/35, 62.8%) and visual field deficits (13/35, 37.1%). Tumor recurrence was detected in 9 male and 11 female patients, with a mean recurrence time of 35.8 months (range: 10–68 months). Most patients (18/20, 90%) experienced recurrence within 5 years, with a mean time of 32.4 months (range: 10–55 months). Tumor regrowth was observed in 4 male and 11 female patients, with a mean regrowth time of 25.4 months (range: 6–58 months). Patient characteristics are summarized in Table 1; there was no difference found with respect to gender, age, tumor size, or invasion among groups.

**Table 1:** The patient characteristics of recurrence/regrowth group

Feature	Recurrence			
	Primary	Recurrence	Primary	Regrowth
Number	20	20	15	15
Age (year)				
Range	20–57	21–63	21–59	22–62
Median	37	41	38	41
Mean	37.55	41.45	39.4	41.7
Sex				
Male	9	9	4	4
Female	11	11	11	11
Head ache	14	10	8	8
Diminution of vision	12	8	11	7
Visual field defect	7	4	6	7
Incidental/reexamine	2	3	2	3
Knosp grading				
Grade1	5	4	0	0
Grade2	7	9	5	3
Grade3a	4	3	1	1
Grade3b	4	1	2	3
Grade4	0	3	7	8
Invasive				
Yes	7	5	10	11
No	13	15	5	4
Volume (cm^3^)				
Range	1.144–19.06	2.700–21.00	1.092–51.28	1.825–36.52
Median	7.182	6.000	14.18	10.50
Mean	8.540	7.637	16.46	12.92
Recurrence/regrowth time (m)				
Range		10–68		6–58
Median		35		27
Mean		35.8		25.4

### HMGA1, Ki67, MDM2, and RB expression in recurrent NFPA

**Fig. 1:**
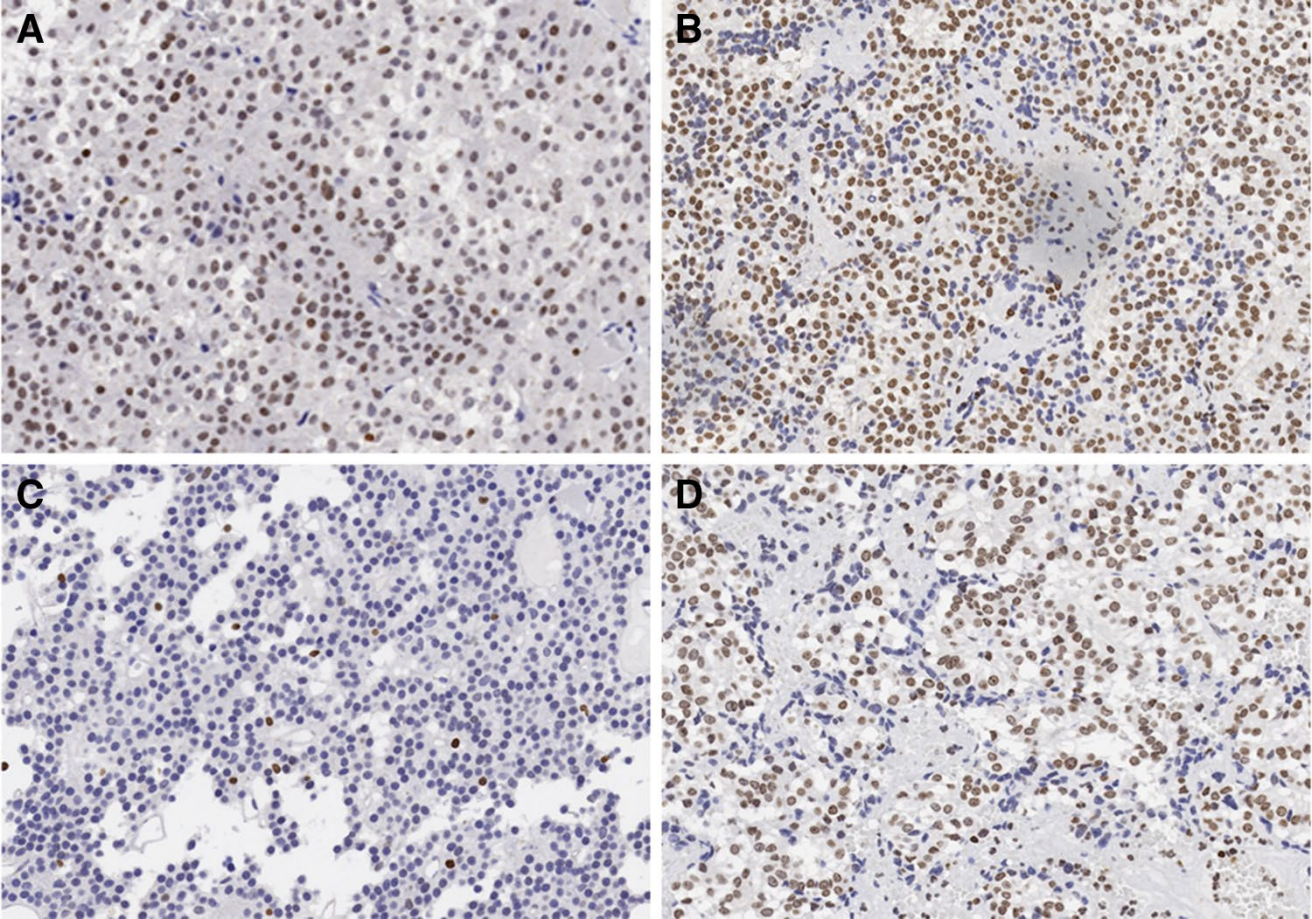
The expression level of HMGA1. **a** In the primary tumor specimens. **b** In the recurrence tumor specimens. **c** In the residual tumor specimens. **d** In the regrowth tumor specimens. ×200

**Fig. 2:**
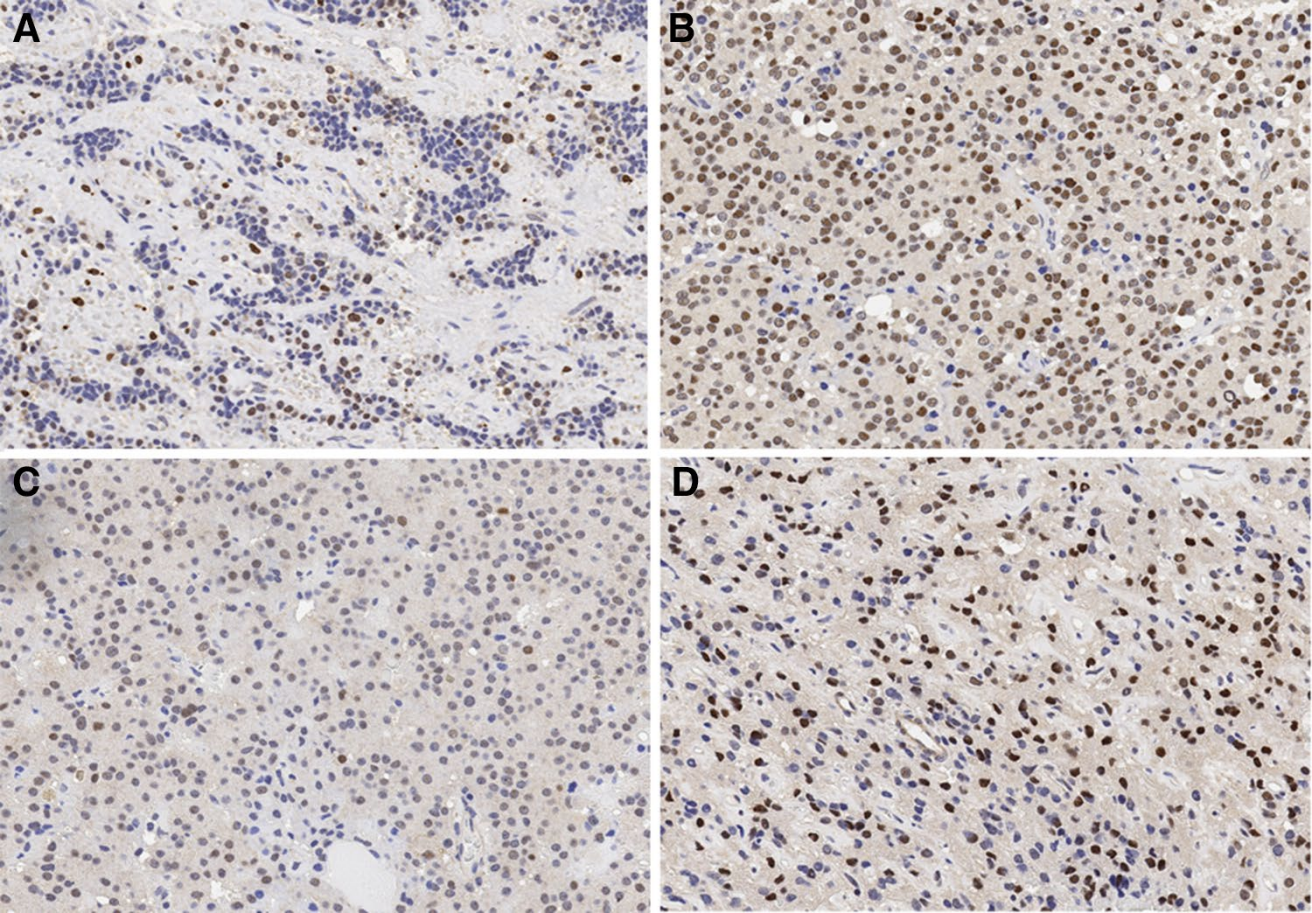
The expression level of MDM2. **a** In the primary tumor specimens. **b** In the recurrence tumor specimens. **c** In the residual tumor specimens. **d** In the regrowth tumor specimens. ×200

Samples were scored for nuclear expression of HMGA1, Ki67, MDM2 and RB. Of the 20 samples, nine were HMGA1-positive, with Hscores of 43.75 and 100 for primary and recurrent tumors, respectively (χ^2^ = 5.227, P = 0.022; Fig. 1a, b). There were 11/20 MDM2-positive cases, with H-scores of 65.45 and 170 for primary and recurrent tumors, respectively (χ^2^ = 8.533, P = 0.003; Fig. 2a, b). There were no statistically significant differences in rates of RB positivity between the primary and recurrent tumor groups, although the H-score was higher in the latter (Table 2).Ki-67 is a marker for proliferating cells in neoplastic lesions; the Ki-67 LI was found to be in the range of 0–12.4% for moderate immunoreactivity (2+), with mean values of 2.76 and 4.09% for primary and recurrent groups, respectively.

**Table 2:** The HMGA1, MDM2, Ki67 and RB level in recurrence group

Gene	HMGA1			MDM2			Ki67			RB		
Classification	(−)	(+)	H-Score	(−)	(+)	H-Score	(−)	(+)	H-Score	(−)	(+)	H-Score
Primary	11	9	43.75	9	11	65.45	19	1	2.76	3	17	80.43
Recurrence	4	16	100	1	19	170	17	3	4.09	0	20	138

### HMGA1, Ki67, MDM2, and RB expression in regrowth NFPA

Among the 15 cases of regrowth NFPA, four were HMGA1-positive with H-scores of 16.84 and 76.67 for residual and regrowth tumors, respectively (χ^2^ = 8.571, P = 0.003; Fig. 1c, d). There were nine MDM2-positive cases, with H-scores of 71.58 and 146 for residual and regrowth tumors, respectively (χ^2^ = 7.5, P = 0.006; Fig. 2c, d). There was no statistically significant difference in the rates of RB positivity between the primary and regrowth tumor groups, although the H-score was slightly higher in the latter (Table 3). The mean Ki-67 LI was 2.52 and 3.43 in the residual and regrowth groups, respectively. To assess the significance of HMGA1 and MDM2 over expression in NFPA, we examined the relationship between HMGA1 and MDM2 immunoreactivity and clinicopathologic features. There were no significant differences in terms of gender, age, tumor size, and invasion between primary tumor and recurrence/regrowth groups.

**Table 3:** The HMGA1, MDM2,Ki67 and RB level in regrowth group

Gene	HMGA1			MDM2			Ki67			RB		
Classification	(−)	(+)	H-score	(−)	(+)	H-Score	(−)	(+)	H-Score	(−)	(+)	H-score
Residual	11	4	16.84	6	9	71.58	14	1	2.52	1	14	91.05
Regrowth	3	12	76.67	0	15	146	14	1	3,43	1	14	126.7

## Discussion

Surgery is still the first and only treatment option for treating NFPA, and can rapidly improve clinical symptoms, including the headache, visual disturbance, and visual field deficits observed in this study. Visual disturbances due to compression of the optic apparatus are common, occurring in 30.8–67.8% of cases [21, 22], whereas visual field deficits related to compression of the optic chiasm are observed in 60.8% of patients [23]. The frequency of headaches varies between 9.7 and 60.8% [21–23]. In our study, 90% of patients showed recurrence within 5 years, consistent with a previous report that the majority of cases show recurrence between 1 and 5 years after surgery [24].

This is the first study to compare the expression of Ki-67, MDM2, HMGA1, and RB in paired primary and recurrent/regrowth NFPA specimens. HMGA, MDM2, and RB regulate different points of the cell cycle and therefore play critical roles in pituitary cell proliferation and pituitary adenoma development. We found no difference in the levels of these proteins between the recurrence and regrowth groups, suggesting a higher risk associated with non-radical excision of recurrent adenoma. A microsurgical transsphenoidal approach was used in most patients in this study, which often does not include the whole medial cavernous sinus wall; therefore, patients should be closely monitored for tumor recurrence [25], although post-operative residual adenoma is not an independent predictor of recurrence [26].Modern molecular genetic techniques have already exposed that a pituitary adenoma grow out of a single cell. So sometimes it is impossible for radical resection of some NFPAs in histological level, and the remnant tumor can potentially regrow since residual cells retain their ability to proliferate.

### Prognostic significance of HMGA1

The HMGA protein family includes HMGA1 and the closely related HMGA2 protein, which are non-histone chromosomal proteins that target the minor groove of AT-rich DNA strands through their adenine/thymine-binding motifs [27].HMGA overexpression is a feature of cancers of the colon, rectum, breast, pancreas, ovary, lung, esophagus, and testis, among others, and can be used to predict patient prognosis and drug response [28]. HMGA overexpression reflects the dysregulation of cell cycle-related proteins in pituitary adenomas and is associated with tumor invasion through interaction with the RB-E2F1 pathway [12]. It has been reported that HMGA proteins upregulate cyclin B2 expression, which is correlated with human pituitary tumorigenesis [29]. However, there is no obvious relationship between the HMGA-1 and tumor regrowth [30]. HMGA1 is a therapeutic target in pancreatic cancer [31]. Here we found that HMGA1 was upregulated in recurrent and regrowthtumors as compared to primary tumors, suggesting that it plays a significant role in NFPA progression.

### Prognostic significance of Ki-67

Ki-67 is an immunohistochemical marker routinely used in pituitary adenomas, but its prognostic significance is debated [18]. Previous studies have reported Ki-67 positivity rates of 2.7–15% [32–34]. Several studies have explored the possibility of using Ki-67 as a prognostic marker of tumor recurrence or regrowth [30]. One study reported that tumorigenesis was correlated with a Ki-67 LI >2% [40]; others have demonstrated that an LI >2.2% was associated with residual tumor growth [30] or that an LI >3% was a strong prognostic factor for pituitary adenoma recurrence/progression [35]. However, some investigators have found no correlation between Ki-67 expression and post-operative tumor behavior [36]. We found a Ki-67 LI of 0–12.4% associated with moderate staining intensity (2+) with no difference between primary/residual and recurrent/regrowth adenomas. The elevated Ki-67 index revealed a strong tendency which suggests that Ki-67 plays some extent role in adenoma progression. Some authors had a similar conclusion. Micko revealed a strong tendency between invasive and non-invasive adenoma, and no statistically significant correlation to higher MIB-1 in invasive cases [37]. Indeed, there was no association between Ki-67 LI and Knosp classification, which were similar in adenomas with total and partial surgical removal [38].

### Prognostic significance of MDM2

MDM2 is an oncogene that promotes tumor transformation, invasion, and metastasis in a p53-independent manner [39]. MDM2 negatively regulates p53 via various mechanisms such as cell cycle control, genome stability, apoptosis, and tumor neoangiogenesis by ubiquitination, transcription factor activation, and regulation of mRNA stability [40].MDM2 is overexpressed in various human tumors, including sarcoma, leukemia, breast carcinoma, melanoma, and glioblastoma [41]. A recent study detected MDM2 nuclear expression in 21.3% of malignant pleural mesothelioma patients, which was correlated with worse prognosis [42]. The Nutlin analog RO5503781 targets the p53-binding site on MDM2 protein and increases its potency [43].In this study, MDM2 was detected in 55% of primary and 95% of recurrent NFPA specimens, while MDM2 positivity was observed in 60% of primaryand in 100% of regrowth specimens. The higher expression MDM2 in recurrent and regrowthtumors indicate an associated with NFPA progression.

### Prognostic significance of RB

The RB tumor suppressor directly and indirectly modulates tumor development as a negative regulator of the cell cycle via interaction with members of the E2F family [44]. In fact, RB inhibits both cell cycle progression and apoptosis [45]. Although we found no difference in RB expression among groups, all specimens showed positive RB expression, suggesting that RB is involved in the development of NFPA.

## Conclusions

In this study, we found that NFPA recurrence and regrowth behave in a similar fashion. HGMA1 and MDM2 can potentially serve as therapeutic targets or biomarkers for NFPA, whereas. Ki-67 is an important prognostic marker of NFPA progression. Additional studies with a larger study population are needed to confirm these findings.
